# Modeling and analysis of long booster clustered launch-vehicle

**DOI:** 10.1038/s41598-020-58173-6

**Published:** 2020-01-31

**Authors:** Jiangtao Xu, Ya Yang, Bangsheng Fu, Dewei Zhang

**Affiliations:** 1 Department of Aerospace Engineering, Harbin Engineering University, Harbin, 150001 Heilongjiang Province China; 2 College of Electronic Information, Zhongyuan Institute of Technology, Zhengzhou, 450007 Henan Province China

**Keywords:** Engineering, Aerospace engineering

## Abstract

According to the characteristics of complex spatial mode of long booster clustered launch vehicle, it is not suitable to derive the dynamic equations based on the simplified model of single beam considering only core rocket. In this paper, the long booster clustered launch-vehicle is simplified to a multi-beam model considering core and boosters. Then, due to the high proportion of the liquid propellant in the total mass of the launch vehicle, on the basis of the multi-beam model, a dynamic equation considering the elastic deformation and the liquid sloshing is established. The dynamic characteristics of the launch-vehicle and the interaction between liquid sloshing and aeroelasticity are studied by simulation. Based on the multi-beam model, the connection types between the core and the booster rocket are simplified to an elastic connection. The influence of the connection on the dynamic characteristics of the launch-vehicle is discussed. The results demonstrate that the multi-beam model can more fully reflect the dynamic characteristics of the long booster clustered launch-vehicle, and the connection form between the core and the boosters also has an important effect on the liquid sloshing and the elastic deformation.

## Introduction

In order to improve the carrying capacity and put more payloads into space, the world’s major aerospace countries have developed and used a large long booster clustered launch-vehicle. With the development of the aerospace industry, the length of the booster rocket is sfeadly increasing^1^. From the study of short booster clustered launch-vehicles, in the frequency band associated with the attitude control system, the booster basically acts as a follower. The booster itself does not exhibit significant elastic deformation^2^, and a small amount of local deformation of the booster does not attract attention in engineering. The main deformation modes of short boost rocket are basically core stage rocket deformation modes. Therefore, the short booster rocket can be simplified into a single beam model^3–5^. Wu^6,7^ regarded a slender vehicle as a free-free beam. The mechanism of slender vehicle instability under direction-controlled thrusts was studied by the finite element method, and the relationship between structural stability and elastic modes was analyzed. Trikha^8,9^ considered the coupling of rigid, longitudinal and lateral vibration modes and established a dynamic model for analyzing the stability of slender vehicles under thrust and aerodynamic forces. However, with the increase of the length of the booster, the aerodynamic force acting on the boost rocket will increase and the natural frequency will decrease. The booster will produce a large local deformation, and then affecting the dynamic characteristics of the core rocket. Therefore, the dynamic equation based on the single beam model is not sufficient.

In general, launch vehicles need to carry large amounts of liquid fuel. The liquid propellant is about 90 percent of the take-off mass of the launch vehicle. The periodic force produced by liquid sloshing not only results in structural damage of the launch vehicle, but also is coupled with the rigid body motion and the elastic vibration. This can leads to the failure of the attitude control system. Therefore, the propellant dynamics model has an important influence of the study on the structural dynamic characteristics of the launch vehicle^10,11^. R. Playter^12^ established a nonlinear dynamics model considering the significant coupling between fuel slosh, engine gimballing and vehicle rigid and flexural motion. This paper presents a unified analysis of the coupled non- linear dynamics of the rigid booster vehicle, flex modes, slosh masses, and engines. O. Bayle^13^ described the propellant sloshing phenomenon of spacecraft and its influence on the flight performance. In this paper, Computational Fluid Dynamics (CFD) simulation of the liquid fuel sloshing are presented, and their results are compared with the equivalent mechanics model based on spring. Kailash^14^ and Ashivni^15^ established the kinetic equation of the rocket plane motion by using the sloshing equivalent mechanics model and the rigid body model. At the same time, the influence of the engine gimbal torque on the stability of rocket attitude motion was analyzed. As an extension to the additional mass method and the mass effect of the longitudinal and lateral propellant in the liquid filled tank, Pan^16,17^ established a dynamic model of the longitudinal, lateral and torsion of the launch vehicle.

In this paper, according to the characteristics of complex spatial mode of long booster clustered launch vehicle, the vehicle is simplified into a multi-beam model. Based on the hybrid coordinate method and the Lagrange energy method, the dynamic equation considering aerodynamic force and thrust is established. Then, on the basis of the beam system model, the dynamic model which considering the effect of liquid sloshing is established. The coupling effects of liquid sloshing and elastic deformation are studied. Further more, the simulation results are compared with the traditional singal beam model. Finally, based on the above results, the influence of the connection between core and booster on liquid sloshing and elastic deformation is analysed.

## Beam System Model and Dynamic Modeling

**Figure 1:**
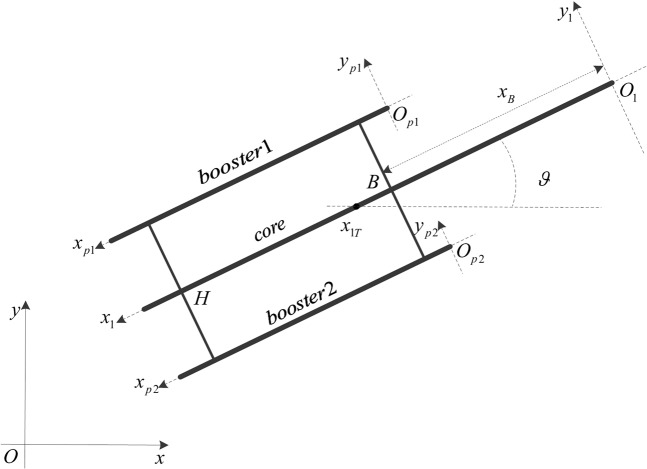
Multi-beam model of launch vehicle.

The long booster clustered launch vehicle is simplified into a muti-beam model as shown in Fig. 1. The connection point between core and booster is rigid connection. No elastic deformation occurs at the connection point of booster. The following will be based on this model to derive the launch vehicle pitch plane dynamics equation.

In Fig. 1, *oxy* is the inertial frame of the origin at the launching point. The direction *ox* and *oy* are shown in Fig. 1. *o*_1_*x*_1_*y*_1_ is a coordinate system that moves with the launch vehicle, whose origin is the vertex of the core. *o*_*p*1_*x*_*p*1_*y*_*p*1_ and *o*_*p*2_*x*_*p*2_*y*_*p*2_ are moving coordinate systems of the origin fixed at the vertex of booster rockets. *x*_1*T*_ is the position of the center of mass in the *o*_1_*x*_1_*y*_1_. *x*_*B*_ and *x*_*H*_ are, respectively, the distance from the connection point B and H to the origin of the *o*_1_*x*_1_*y*_1_. *ϑ* is the pitch angle.

Toward the modeling of the system, following assumptions are adopted:

1. The elastic deformation of core and boosters is small;
2. The torsional deformation can be ignored;
3. The inertia force caused by the rotation of the earth can be ignored;
4. The change of the center of mass has nothing to do with the elastic deformation.

The velocity components in *oy* are given by

1 $${\dot{y}}_{core}({x}_{1},t)={\dot{y}}_{T}(t)+\left(\dot{\vartheta }(t)({x}_{1T}-{x}_{1})+\mathop{\sum }\limits_{i=1}^{n}{\dot{S}}_{i}(t){f}_{i}({x}_{1})\right)\cos \,\vartheta $$

where, ${\dot{y}}_{T}(t)$ is the velocity of the center of mass in the *oy* direction. $\mathop{\sum }\limits_{i=1}^{n}{\dot{S}}_{i}(t){f}_{i}({x}_{1})$ is the elastic vibration velocity. *f*_*i*_(*x*) is the natural mode of elastic vibration, and *S*_*i*_(*t*) is the generalized coordinates of elastic vibration.

Then, the velocity of the booster1 in the *oy* direction is deduced as follows.

2 $${\dot{y}}_{booster1}({x}_{p1},t)={\dot{y}}_{T}(t)+\left\{\begin{array}{c}\dot{\vartheta }(t)({x}_{1T}-{x}_{B}-{x}_{p1})+\mathop{\sum }\limits_{i=1}^{n}{\dot{K}}_{p1i}(t){\Phi }_{p1i}({x}_{p1})\\ +\,\mathop{\sum }\limits_{i=1}^{n}{\dot{S}}_{i}(t)[{f}_{i}({x}_{H})\frac{{x}_{p1}}{{a}_{1}}+{f}_{i}({x}_{B})(1-\frac{{x}_{p1}}{{a}_{1}})]\end{array}\right\}\cos \,\vartheta $$

where, ${K}_{p1i}(t)$ represents the generalized coordinates of the booster1 in the *o*_*p*1_*x*_*p*1_*y*_*p*1_ coordinate system. ${\Phi }_{p1i}(x)$ is the natural vibration modes of the booster1. *a*_1_ is the length of the booster1. The velocity of the booster1 can also be obtained in the similar way.

3 $${\dot{y}}_{booster2}({x}_{p2},t)={\dot{y}}_{T}(t)+\left\{\begin{array}{c}\dot{\vartheta }(t)({x}_{1T}-{x}_{B}-{x}_{p2})+\mathop{\sum }\limits_{i=1}^{n}{\dot{K}}_{p2i}(t){\Phi }_{p2i}({x}_{p2})\\ +\,\mathop{\sum }\limits_{i=1}^{n}{\dot{S}}_{i}(t)[{f}_{i}({x}_{H})\frac{{x}_{p2}}{{a}_{2}}+{f}_{i}({x}_{B})(1-\frac{{x}_{p2}}{{a}_{2}})]\end{array}\right\}\cos \,\vartheta $$

The velocity of core and boosters in the direction of *oy* is deduced as follows.

4 $${\dot{x}}_{core}({x}_{1},t)={\dot{x}}_{T}(t)-\left(\dot{\vartheta }(t)({x}_{1T}-{x}_{1})+\mathop{\sum }\limits_{i=1}^{n}{\dot{S}}_{i}(t){f}_{i}({x}_{1})\right)\sin \,\vartheta $$

5 $${\dot{x}}_{booster1}({x}_{p1},t)={\dot{x}}_{T}(t)-\left\{\begin{array}{c}\dot{\vartheta }(t)({x}_{1T}-{x}_{B}-{x}_{p1})+\mathop{\sum }\limits_{i=1}^{n}{\dot{K}}_{p1i}(t){\Phi }_{p1i}({x}_{p})\\ +\,\mathop{\sum }\limits_{i=1}^{n}{\dot{S}}_{i}(t)[{f}_{i}({x}_{H})\frac{{x}_{p1}}{{a}_{1}}+{f}_{i}({x}_{B})(1-\frac{{x}_{p1}}{{a}_{1}})]\end{array}\right\}\sin \,\vartheta $$

6 $${\dot{x}}_{booster2}({x}_{p2},t)={\dot{x}}_{T}(t)-\left\{\begin{array}{c}\dot{\vartheta }(t)({x}_{1T}-{x}_{B}-{x}_{p1})+\mathop{\sum }\limits_{i=1}^{n}{\dot{K}}_{p2i}(t){\Phi }_{p2i}({x}_{p})\\ +\,\mathop{\sum }\limits_{i=1}^{n}{\dot{S}}_{i}(t)[{f}_{i}({x}_{H})\frac{{x}_{p2}}{{a}_{2}}+{f}_{i}({x}_{B})(1-\frac{{x}_{p2}}{{a}_{2}})]\end{array}\right\}\sin \,\vartheta $$

So we can get the kinetic energy of the core, booster1 and booster2 respectively is

7 $${T}_{core}=\frac{1}{2}{\int }_{0}^{l}{m}_{c}({x}_{1})({\dot{y}}_{core}^{2}({x}_{1},t)+{\dot{x}}_{core}^{2}({x}_{1},t))d{x}_{1}$$

8 $${T}_{booster1}=\frac{1}{2}{\int }_{0}^{{a}_{1}}{m}_{booster1}({x}_{p1})({\dot{y}}_{booster1}^{2}({x}_{p1},t)+{\dot{x}}_{booster1}^{2}({x}_{p1},t))d{x}_{p1}$$

9 $${T}_{booster2}=\frac{1}{2}{\int }_{0}^{{a}_{2}}{m}_{booster2}({x}_{p2})({\dot{y}}_{booster2}^{2}({x}_{p2},t)+{\dot{x}}_{booster2}^{2}({x}_{p2},t))d{x}_{p2}$$

The total kinetic energy T is

10 $$T={T}_{c}+{T}_{booster1}+{T}_{booster2}$$

The following will derive the potential energy expression of the launch vehicle. The potential energy of any elastic system is mainly caused by the strain that accumulates in its components. According to the specific problems and the actual situation, the strain energy expression of the variable cross-section bar is used to obtain the strain energy. Then the potential expression of the core and booster rocket respectively is

11 $${U}_{core}=\frac{1}{2}{\int }_{0}^{l}{E}_{core}({x}_{1}){I}_{core}({x}_{1}){\left[\frac{{\partial }^{2}{Y}_{core}({x}_{1},t)}{\partial {x}_{1}^{2}}\right]}^{2}d{x}_{1}$$

12 $${U}_{booster1}=\frac{1}{2}{\int }_{0}^{{a}_{1}}{E}_{booster1}({x}_{p1}){I}_{booster1}({x}_{p1}){\left[\frac{{\partial }^{2}{Y}_{booster1}({x}_{p1},t)}{\partial {x}_{p1}^{2}}\right]}^{2}d{x}_{p1}$$

13 $${U}_{booster1}=\frac{1}{2}{\int }_{0}^{{a}_{1}}{E}_{booster1}({x}_{p1}){I}_{booster1}({x}_{p1}){\left[\frac{{\partial }^{2}{Y}_{booster1}({x}_{p1},t)}{\partial {x}_{p1}^{2}}\right]}^{2}d{x}_{p1}$$

where, *E*_*core*_(*x*_1_), *E*_*booster*1_(*x*_*p*1_) and *E*_*booster*2_(*x*_*p*2_) are respectively the elastic modulus of core, booster1 and booster2. *I*_*core*_(*x*_1_), *I*_*booster*1_(*x*_*p*1_), and *I*_*booster*1_(*x*_*p*2_) are the moment of inertia. *I*_*core*_(*x*_1_, *t*), *Y*_*booster*1_(*x*_*p*1_) and *Y*_*booster*2_(*x*_*p*2_) are respectively the elastic displacement of core, booster1 and booster2. So, the total potential energy is

14 $$U={U}_{core}+{U}_{booster1}+{U}_{booster2}$$

We choose *x*(*t*), *y*(*t*), *ϑ*(*t*), *S*_*i*_(*t*) and *K*_*pi*_(*t*)(*p* = 1, 2) as generalized coordinates. Then, the generalized forces corresponding to these generalized coordinates are obtained by the principle of virtual work.

15 $${Q}_{x}=(P-{C}_{x}q{S}_{m})\cos \,\vartheta -\left(\begin{array}{c}q{S}_{m}{C}_{y}^{\vartheta }\vartheta \\ +\,q{S}_{m}\left(\begin{array}{c}{\int }_{0}^{l}\frac{\partial {C}_{y}^{\vartheta }}{\partial {x}_{1}}\frac{\partial {Y}_{c}({x}_{1},t)}{\partial {x}_{1}}d{x}_{1}\\ +\mathop{\sum }\limits_{p=1}^{2}{\int }_{0}^{a}\frac{\partial {C}_{y}^{\vartheta }}{\partial {x}_{p}}\frac{\partial {Y}_{p}({x}_{p},t)}{\partial {x}_{p}}d{x}_{p}\end{array}\right)\end{array}\right)\sin \,\vartheta $$

16 $${Q}_{y}=(P-{C}_{x}q{S}_{m})\sin \,\vartheta -\left(\begin{array}{c}q{S}_{m}{C}_{y}^{\vartheta }\vartheta \\ +\,q{S}_{m}\left(\begin{array}{c}{\int }_{0}^{l}\frac{\partial {C}_{y}^{\vartheta }}{\partial {x}_{1}}\frac{\partial {Y}_{c}({x}_{1},t)}{\partial {x}_{1}}d{x}_{1}\\ +\mathop{\sum }\limits_{p=1}^{2}{\int }_{0}^{a}\frac{\partial {C}_{y}^{\vartheta }}{\partial {x}_{p}}\frac{\partial {Y}_{p}({x}_{p},t)}{\partial {x}_{p}}d{x}_{p}\end{array}\right)\end{array}\right)\cos \,\vartheta $$

17 $${Q}_{\vartheta }=q{S}_{m}{C}_{y}^{\vartheta }\vartheta ({x}_{1T}-{x}_{y})+q{S}_{m}\left(\begin{array}{c}{\int }_{0}^{l}\frac{\partial {C}_{y}^{\vartheta }}{\partial {x}_{1}}({x}_{1T}-{x}_{1})\frac{\partial {Y}_{c}({x}_{1},t)}{\partial {x}_{1}}d{x}_{1}\\ +\,\mathop{\sum }\limits_{p=1}^{2}{\int }_{0}^{a}\frac{\partial {C}_{y}^{\vartheta }}{\partial {x}_{p}}({x}_{1T}-{x}_{p})\frac{\partial {Y}_{p}({x}_{p},t)}{\partial {x}_{p}}d{x}_{p}\end{array}\right)$$

18 $$\begin{array}{c}{Q}_{{S}_{i}}=q{S}_{m}\left({\int }_{0}^{l}\frac{\partial {C}_{y}^{\vartheta }}{\partial {x}_{1}}\frac{\partial {Y}_{c}({x}_{1},t)}{\partial {x}_{1}}d{x}_{1}\right){f}_{i}(x)\\ \,+\,q{S}_{m}\left({\int }_{0}^{l}\frac{\partial {C}_{y}^{\vartheta }}{\partial {x}_{1}}({x}_{1T}-{x}_{1})\frac{\partial {Y}_{c}({x}_{1},t)}{\partial {x}_{1}}d{x}_{1}\right)\frac{\partial {f}_{i}(x)}{\partial x}\end{array}$$

19 $$\begin{array}{c}{Q}_{{K}_{pi}}=q{S}_{pm}\left(\mathop{\sum }\limits_{p=1}^{2}{\int }_{0}^{a}\frac{\partial {C}_{y}^{\vartheta }}{\partial {x}_{p}}\frac{\partial {Y}_{p}({x}_{p},t)}{\partial {x}_{p}}d{x}_{p}\right){\Phi }_{pi}(x)\\ \,+\,q{S}_{pm}\left(\mathop{\sum }\limits_{p=1}^{2}{\int }_{0}^{a}\frac{\partial {C}_{y}^{\vartheta }}{\partial {x}_{p}}({x}_{1T}-{x}_{p})\frac{\partial {Y}_{p}({x}_{p},t)}{\partial {x}_{p}}d{x}_{p}\right)\frac{\partial {\Phi }_{pi}(x)}{\partial x}\end{array}$$

In the formula, *P* is engine thrust. *C*_*x*_ is the aerodynamic coefficient. *q* is the dynamic pressure. *S*_*m*_ and *S*_*pm*_ are characteristic area. ${C}_{y}^{\vartheta }$ is the derivative of lift coefficient to pitch angle. *x*_*y*_ is the center of pressure to the origin of the *o*_1_*x*_1_*y*_1_.

According to the constraint between the core and the booster, the corresponding boundary condition is

20 $$\begin{array}{llllll}{\mathop{\sum }\limits_{i=1}^{n}{K}_{p2i}(t){\Phi }_{p1i}({x}_{p})|}_{{x}_{p}={x}_{p1B}} & = & 0, & {\mathop{\sum }\limits_{i=1}^{n}{K}_{p2i}(t){\Phi }_{p1i}({x}_{p})|}_{{x}_{p}={x}_{p1H}} & = & 0\\ {\mathop{\sum }\limits_{i=1}^{n}{K}_{p2i}(t)\frac{\partial {\Phi }_{p1i}({x}_{p})}{\partial {x}_{p}}|}_{{x}_{p}={x}_{p1B}} & = & 0, & {\mathop{\sum }\limits_{i=1}^{n}{K}_{p2i}(t)\frac{\partial {\Phi }_{p1i}({x}_{p})}{\partial {x}_{p}}|}_{{x}_{p}={x}_{p1H}} & = & 0\\ {\mathop{\sum }\limits_{i=1}^{n}{K}_{p2i}(t){\Phi }_{p2i}({x}_{p})|}_{{x}_{p}={x}_{p2B}} & = & 0, & {\mathop{\sum }\limits_{i=1}^{n}{K}_{p2i}(t){\Phi }_{p2i}({x}_{p})|}_{{x}_{p}={x}_{p2H}} & = & 0\\ {\mathop{\sum }\limits_{i=1}^{n}{K}_{p2i}(t)\frac{\partial {\Phi }_{p2i}({x}_{p})}{\partial {x}_{p}}|}_{{x}_{p}={x}_{p2B}} & = & 0, & {\mathop{\sum }\limits_{i=1}^{n}{K}_{p2i}(t)\frac{\partial {\Phi }_{p2i}({x}_{p})}{\partial {x}_{p}}|}_{{x}_{p}={x}_{p2H}} & = & 0\end{array}$$

The Lagrangian equation is

21 $$\frac{d}{dt}\left(\frac{\partial T}{\partial {\dot{q}}_{j}}\right)-\frac{\partial T}{\partial {q}_{j}}+\frac{\partial U}{\partial {q}_{j}}={Q}_{j}$$

By introducing Eqs. (1–19) into Eq. (21), the dynamic model of long booster clustered launch vehicle based on the multi-beam model can be obtained.

## Dynamic Modeling of Liquid Sloshing

In this paper, the equivalent mechanical model is used to study the sloshing of liquid fuel. The equivalent mechanical model is more efficient in the study of liquid sloshing. Kana^18^ used the equivalent spring-mass system to represent the transverse and longitudinal modes of liquid sloshing, and the coupled liquidstructural dynamics of a typical space shuttle configuration—a parallel-stage design is analysed. Sayar^19^ combined the spring-damper-mass system which represents the vibration of the structure and the simple pendulum system which represents the motion of the liquid to simulate the interaction between the solid structure and the liquid propellant, and the coupling equation is established. Nichkawde^20^ applied the equivalent simple pendulum model to analyze the stability of rigid launch vehicle in plane flight. Unruh^21^ used the “circle fit” method to estimate the simple pendulum model parameters. The spring-damper-mass model and the simple pendulum model can accurately reflect the liquid sloshing characteristics under the assumption that the liquid sloshing amplitude is small. However, the derivation process of spring-damper-mass model is relatively simple. Therefore, the spring mass model is adopted in this paper.

**Figure 2:**
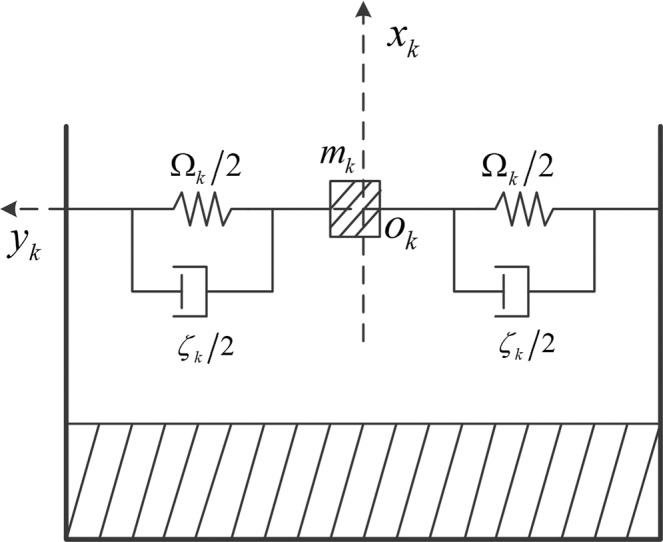
Simplified model of liquid sloshing.

It is assumed that the sloshing amplitude of liquid fuel in the tank is small. Then the fuel tank is simplified into the form of Fig. 2 and the sloshing equation based on this simplified model is deduced.

In Fig. 2, *m*_*k*_ is the sloshing mass of the kth tank. Ω_*k*_ is the equivalent spring constant. ${\zeta }_{k}$ is sloshing damping coefficient. *o*_*k*_*x*_*k*_*y*_*k*_ is the sloshing coordinate system. The origin is the location of the sloshing mass block at rest. The *x*_*k*_ axis is the symmetry axis of the kth tank. The y-axis is in the pitch plane and perpendicular to the x-axis. Then the sloshing equation is derived. The displacement of sloshing mass in *x* and *y* direction is

22 $${x}_{{m}_{k}}={x}_{T}-\left({y}_{k}+\vartheta ({x}_{1T}-{x}_{n})+\mathop{\sum }\limits_{i=1}^{n}{S}_{i}(t){f}_{i}(x)\right)\sin \,\vartheta $$

23 $${y}_{{m}_{k}}={y}_{T}+\left({y}_{k}+\vartheta ({x}_{1T}-{x}_{n})+\mathop{\sum }\limits_{i=1}^{n}{S}_{i}(t){f}_{i}(x)\right)\cos \,\vartheta $$

The velocity of the sloshing mass is ${\dot{x}}_{{m}_{k}}$, ${\dot{y}}_{{m}_{k}}$, so the kinetic energy is

24 $${T}_{k}=\frac{1}{2}{m}_{k}\left({\dot{x}}_{{m}_{k}}^{2}+{\dot{y}}_{{m}_{k}}^{2}\right)$$

The potential energy of the mass block is

25 $${U}_{k}=\frac{1}{2}{\Omega }_{k}{y}_{k}^{2}$$

The dissipated energy is

26 $${D}_{k}=\frac{1}{2}{\zeta }_{p}{\dot{y}}_{k}^{2}$$

The Lagrangian equation considering the dissipated energy is

27 $$\frac{d}{dt}\left(\frac{\partial T}{\partial {\dot{q}}_{j}}\right)-\frac{\partial T}{\partial {q}_{j}}+\frac{\partial U}{\partial {q}_{j}}+\frac{\partial D}{\partial {\dot{q}}_{j}}={Q}_{j}$$

By introducing the total kinetic energy, potential energy and dissipation energy into the Lagrangian Eq. (27), we can get the dynamic equation considering the liquid sloshing based on the beam system model.

## The Dynamic Modeling when Considering the Connection Forms Between Core and Boosters

**Figure 3:**
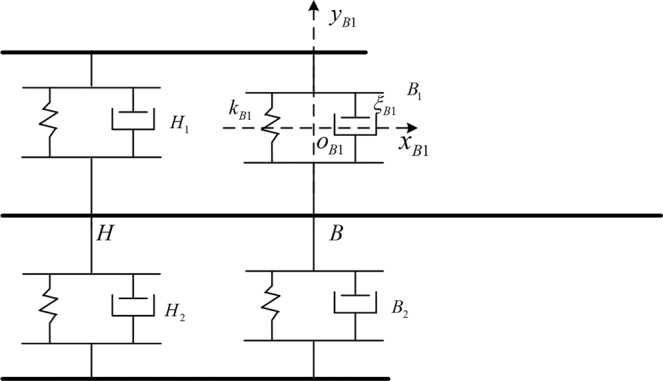
The multi-beam model when considering the connection between core and boosters.

The previous study was based on the assumption that the connection was rigid. The connection itself does not produce a displacement, but this does not fit the actual situation. For this reason we will simplify the connection to the form shown in Fig. 3 and carry out modeling analysis. Under this simplified condition, the booster still has no elastic deformation at the connection point. In Fig. 3, there are four connections, namely *B*_1_, *B*_2_, *H*_1_, and *H*_2_. Take the *B*_1_ connection as an example to introduce the coordinate system and related parameters. The midpoint of the connection is the origin of the *o*_*B*1_*x*_*B*1_*y*_*B*1_ coordinate system. The *x*_*B*1_ axis is parallel to the core axis, and *y*_*B*1_ axis perpendicular to the axis in the pitch plane. *k*_*B*1_ is the equivalent spring constant and ${\xi }_{B1}$ is the equivalent damping coefficient.

To obtain the model, the following assumptions are used:

1. The elastic deformation of connection is small;
2. The deformation of the connection along the axis of the launch vehicle is ignored.

The following describes the dynamics equation when the connection is elastic.

The velocity of booster1 is

28 $${\dot{y}}_{booster1}({x}_{p1},t)={\dot{y}}_{T}(t)+\left\{\begin{array}{c}\dot{\vartheta }(t)({x}_{1T}-{x}_{B}-{x}_{p1})+\mathop{\sum }\limits_{i=1}^{n}{\dot{K}}_{pi}(t){\Phi }_{pi}({x}_{p1})\\ \,+\,\mathop{\sum }\limits_{i=1}^{n}{\dot{S}}_{i}(t)[{f}_{i}({x}_{H})\frac{{x}_{p1}}{{a}_{1}}+{f}_{i}({x}_{B})(1-\frac{{x}_{p1}}{{a}_{1}})]\\ \,+\,{\dot{y}}_{Hp}\frac{{x}_{p1}}{{a}_{1}}+{\dot{y}}_{Bp}(1-\frac{{x}_{p1}}{{a}_{1}})\end{array}\right\}\cos \,\vartheta $$

29 $${\dot{x}}_{booster1}({x}_{p1},t)={\dot{x}}_{T}(t)-\left\{\begin{array}{c}\dot{\vartheta }(t)({x}_{1T}-{x}_{B}-{x}_{p1})+\mathop{\sum }\limits_{i=1}^{n}{\dot{K}}_{pi}(t){\Phi }_{pi}({x}_{p1})\\ \,+\,\mathop{\sum }\limits_{i=1}^{n}{\dot{S}}_{i}(t)[{f}_{i}({x}_{H})\frac{{x}_{p1}}{{a}_{1}}+{f}_{i}({x}_{B})(1-\frac{{x}_{p1}}{{a}_{1}})]\\ \,+\,{\dot{y}}_{Hp}\frac{{x}_{p1}}{{a}_{1}}+{\dot{y}}_{Bp}(1-\frac{{x}_{p1}}{{a}_{1}})\end{array}\right\}\sin \,\vartheta $$

Bring Eqs. (28–29) into the formula (8–9), the kinetic energy of the booster can be obtained in the form of elastic connection.

30 $${U}_{BH}=\mathop{\sum }\limits_{p=1}^{2}\frac{1}{2}{k}_{Bp}{y}_{Bp}^{2}+\mathop{\sum }\limits_{p=1}^{2}\frac{1}{2}{k}_{Hp}{y}_{Hp}^{2}$$

The total energy dissipation is

31 $${D}_{BH}=\mathop{\sum }\limits_{p=1}^{2}\frac{1}{2}{\xi }_{Bp}{\dot{y}}_{Bp}^{2}+\mathop{\sum }\limits_{p=1}^{2}\frac{1}{2}{\xi }_{Hp}{\dot{y}}_{Hp}^{2}$$

If the influence of liquid sloshing is considered, based on the multi-beam model in Fig. 3, the dynamic model of aeroelastic and liquid sloshing is obtained. Due to the complexity of the final dynamic equation, the dynamic equation is not given.

## Simulation Analysis

**Figure 4:**
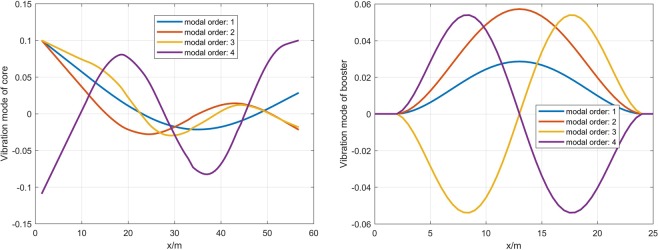
Vibration mode of core and booster.

In order to study the coupling effects of elastic deformation, liquid sloshing and connection modes, some simulations are made in this section. The simulation of this paper depends on the vibration characteristics of the launch vehicle. Therefore, the vibration characteristics of core and booster are shown in Table 1 and Fig. 4.

**Table 1:** Vibration frequency.

modal order	frequency, Hz
1	3.268
2	4.147
3	6.518
4	7.231

It can be seen from Table 1 that the modal frequency of long booster rocket is low and densely distributed. Figure 4 shows the normalized core and booster vibration modes. It can be seen that the booster has a local mode at the same vibration frequency as the core. The influence of these local modes with low frequency can not be ignored.

**Figure 5:**
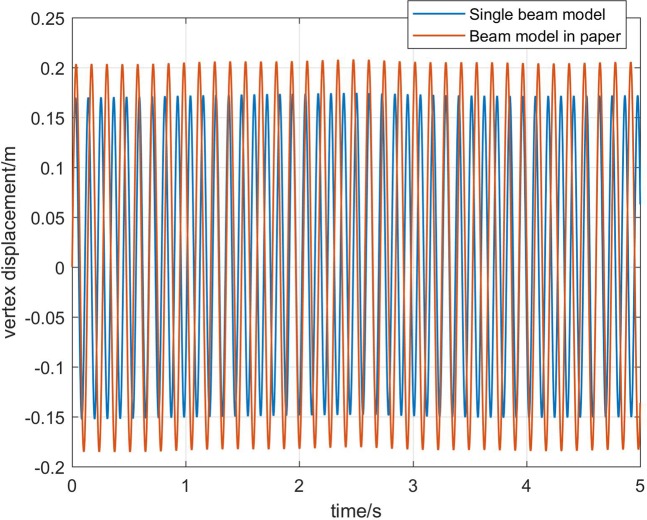
Comparison of the vertex displacement.

Figure 5 shows a comparison of the vertex displacements of core rocket. The displacements calculated by the model in this paper increase by about 20% compared with the traditional model. This shows that the singal beam model without considering booster rockets cannot fully reflect the dynamic characteristics of core rocket. At the same time, this also shows that there is a coupling between the core and the booster rockets. So, the multi-beam model in this paper is valuable for the calculation of flutter critical conditions.

The simulation analysis is carried out considering the influence of liquid sloshing.

**Figure 6:**
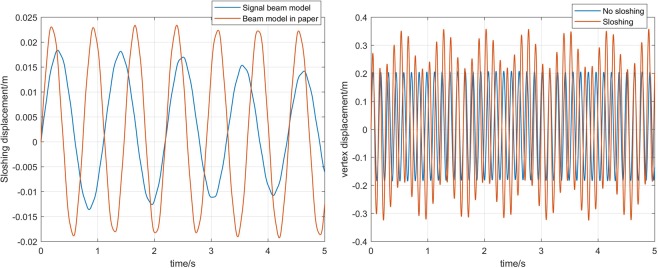
Comparison of displacement.

In the first image of Fig. 6, the displacement of *m*_*k*_ in singal beam model and multi-beam model is compared. As can be observed in the figure, the sloshing displacement calculated by the beam model in this paper is different from the singal beam model in frequency and amplitude. The sloshing amplitude of liquid fuel in the multi-beam model increases by about 25%. The sloshing frequency also increases. The second image in Fig. 6 shows the effect of sloshing on the displacement of the core vertex. It can be seen that when the liquid sloshing is considered, the amplitude of vertex displacement has increased significantly, and the vibration frequency of the vertex is reduced.

According to the previous analysis results, the elastic deformation and sloshing displacement calculated by single beam model are relatively small. So it’s risky to do some related design based on this result.

**Figure 7:**
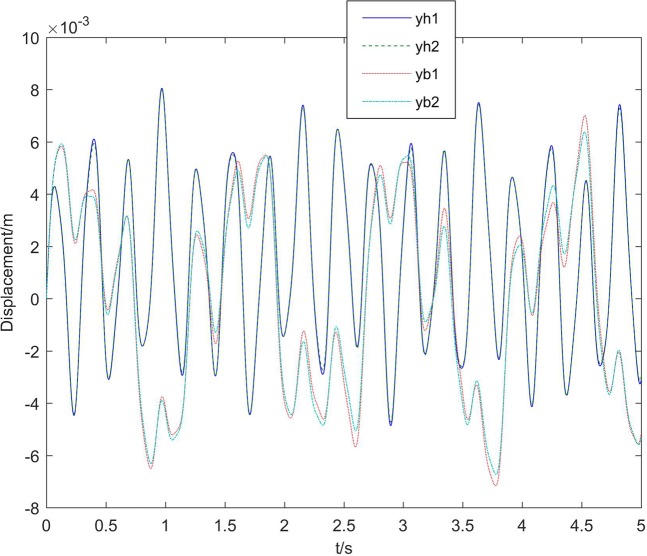
Elastic displacement of the connection.

**Figure 8:**
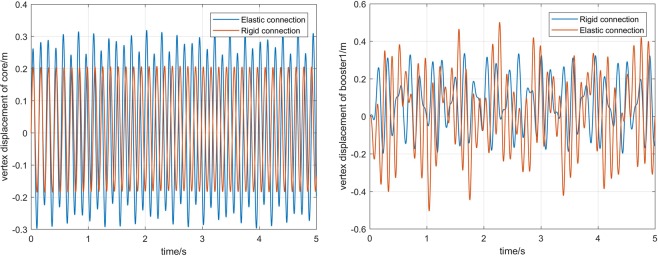
Comparison of displacement under different connection forms.

Next, the simulation analysis of elastic connection is carried out. Figure 7 shows the displacement of the elastic connection between core and boosters, namely *B*_1_, *B*_2_, *H*_1_, and *H*_2_,. Figure 8 shows the comparison of core vertex displacement and booster1 vertex displacement under elastic and rigid connections, respectively. As can be seen from the first image Fig. 8, when the connection is elastic, the vertex displacement of core have some change in frequency and amplitude, and the amplitude increases by about 15%. However, the vibration frequency is reduced a little. So it is necessary to consider not only the beam model, but also the influence of the connection form on the dynamics modeling of the long booster launch vehicle. The last figures in Fig. 8 show the variation of the vertex displacement of booster1. It can be seen that the elastic connection will greatly increase the vibration displacement of the booster, which will affect its flight stability.

**Figure 9:**
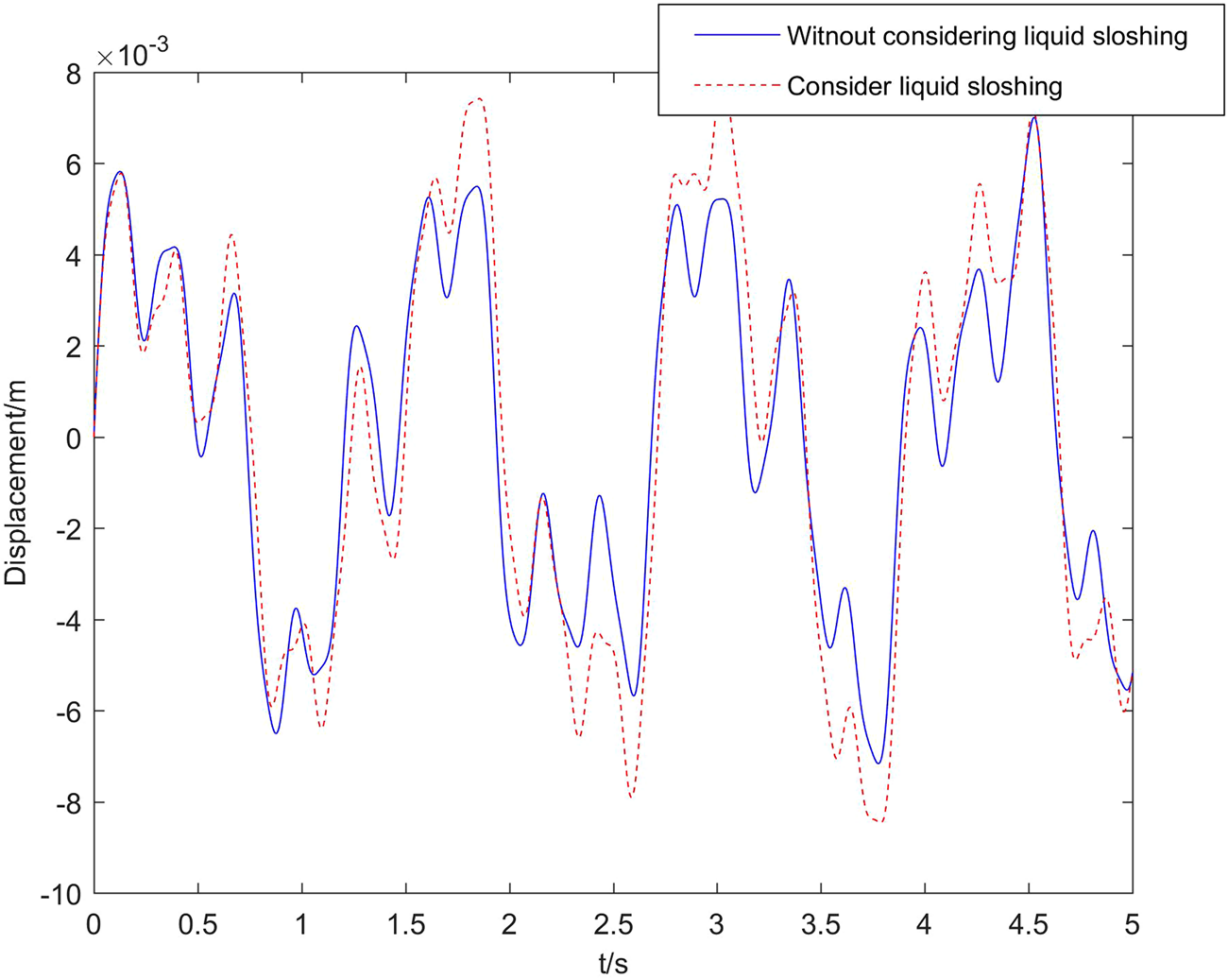
Comparison of elastic displacement of *B*_1_.

**Figure 10:**
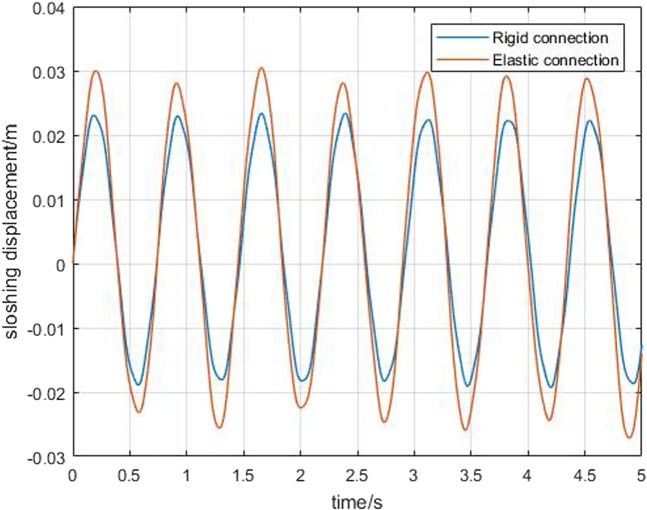
Comparison of sloshing displacement.

**Figure 11:**
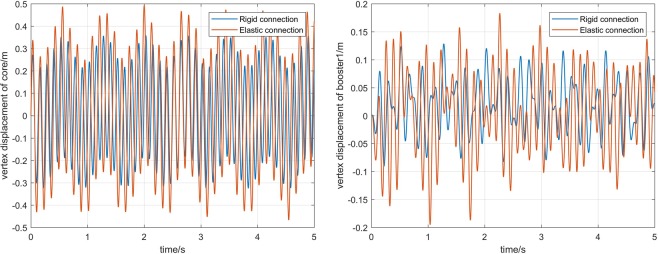
Comparison of the vertex displacement.

Figure 9 is a comparison of the displacement of the *B*_1_ connection with or without considering the sloshing of the liquid. We can get that, when considering the influence of liquid sloshing, the displacement of *B*_1_ connection has changed greatly, and it will affect the elastic displacement. Figure 10 shows the effect of the elastic connection on the liquid sloshing. The frequency of the liquid sloshing does not change much, but the amplitude increases. Figure 11 is the comparison of the vertex displacement of the core and booster1 under different connection forms when considering liquid sloshing, respectively. It can be seen from the Fig. 11 that the vertex displacement of the core does not change much, but the amplitude of the booster1 is greatly increased.

**Figure 12:**
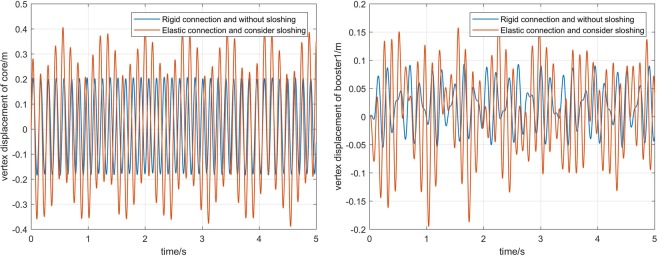
Comparison of the vertex displacement.

The first picture in Fig. 12 shows the contrast of the vertex displacement of the core in multi-beam model with and without considering the elastic connection and the liquid sloshing. It can be seen from the figure that the frequency and amplitude of the vertex displacement of core are changed. The maximum value is increased by 10%, and the minimum value is reduced by 30%. The second picture in Fig. 12 shows the change in the vertex displacement of the boosters. We find that the magnitude of the change is much larger than the core vertex displacement, indicating that liquid sloshing and elastic connections have a significant effect on the booster.

## Summary and Conclusion

With the increase of the length of the booster, the local deformation mode of the booster appears in the low frequency range. The influence of these modes on the dynamic characteristics of the launch vehicle is more and more obvious, and the complexity of the vibration modes of the launch vehicle is also increasing. Therefore, based on the multi-beam model, this paper studies the influence of the local modes of the booster, the connection form between the core and the booster, and liquid fuel sloshing on the dynamic characteristics of the launch vehicle.

Major conclusions are as follows.

1. When modeling based on multi-beam model and considering the elastic deformation of the launch vehicle, the elastic displacement amplitude increases. And compared to the core, the elastic deformation of the boosters cannot be ignored. This shows that there is interaction between the elastic deformation of the core and boosters. For the dynamic analysis, compared with the singal beam model, the multi-beam model can reflect the effect of booster on the vibration characteristics of launch vehicle, and have more reference value.
2. The liquid sloshing leads to the amplitude fluctuation of the elastic displacement of the core and boosters. The results show that there is a serious coupling effect between liquid sloshing and aeroelasticity.
3. The connection forms between the core and the booster have a coupling effect with liquid sloshing and elastic deformation. The elastic connection increases the elastic displacement of the booster rocket and the amplitude of the liquid sloshing.
